# Simultaneous Dual Recordings From Vestibular Hair Cells and Their Calyx Afferents Demonstrate Multiple Modes of Transmission at These Specialized Endings

**DOI:** 10.3389/fneur.2022.891536

**Published:** 2022-07-11

**Authors:** Donatella Contini, Gay R. Holstein, Jonathan J. Art

**Affiliations:** ^1^Department of Anatomy & Cell Biology, University of Illinois College of Medicine, Chicago, IL, United States; ^2^Department of Neurology, Icahn School of Medicine at Mount Sinai, New York, NY, United States; ^3^Department of Neuroscience, Icahn School of Medicine at Mount Sinai, New York, NY, United States

**Keywords:** synaptic transmission, hair cell, calyx, ion accumulation, quantal transmission, ephaptic transmission, resistive coupling, vestibular

## Abstract

In the vestibular periphery, transmission *via* conventional synaptic boutons is supplemented by post-synaptic calyceal endings surrounding Type I hair cells. This review focusses on the multiple modes of communication between these receptors and their enveloping calyces as revealed by simultaneous dual-electrode recordings. Classic orthodromic transmission is accompanied by two forms of bidirectional communication enabled by the extensive cleft between the Type I hair cell and its calyx. The slowest cellular communication low-pass filters the transduction current with a time constant of 10–100 ms: potassium ions accumulate in the synaptic cleft, depolarizing both the hair cell and afferent to potentials greater than necessary for rapid vesicle fusion in the receptor and potentially triggering action potentials in the afferent. On the millisecond timescale, conventional glutamatergic quantal transmission occurs when hair cells are depolarized to potentials sufficient for calcium influx and vesicle fusion. Depolarization also permits a third form of transmission that occurs over tens of microseconds, resulting from the large voltage- and ion-sensitive cleft-facing conductances in both the hair cell and the calyx that are open at their resting potentials. Current flowing out of either the hair cell or the afferent divides into the fraction flowing across the cleft into its cellular partner, and the remainder flowing out of the cleft and into the surrounding fluid compartment. These findings suggest multiple biophysical bases for the extensive repertoire of response dynamics seen in the population of primary vestibular afferent fibers. The results further suggest that evolutionary pressures drive selection for the calyx afferent.

## Introduction

A persistent question in vestibular research is the functional significance of specialized calyx endings present on a subset of primary afferents in vestibular end organs. A majority of afferent and all efferent synapses involving hair cells (HCs) in the labyrinth and cochlea are mediated by conventional bouton endings. While the calyx represents a unique characteristic distinguishing vestibular from auditory sensory epithelia, the function of this morphologically specialized synapse remains obscure. Recent dual simultaneous recordings from HCs and their associated calyx afferents demonstrate that there are three modes of intercellular communication between them. In addition to unidirectional quantal transmission from HCs to afferents, there are two bidirectional forms of transmission. The slower of the two results from potassium flux into the cleft from either HC or afferent over tens of milliseconds, which can elevate [K^+^]_cleft_ from a resting ~8 to >28 mM. The corresponding E_(K)_ for channels facing the cleft are depolarized from −71 to −38 mV. The more rapid form of bidirectional transmission results from HC and afferent channels open near their respective resting potentials. These allow current flowing into the cleft from either HC or afferent to divide between a component flowing out of the cleft near its apex at the neck of the Type I HC and a component flowing across the cleft into the synaptic partner. As yet, there is no compelling evidence that the calyx is uniquely associated with particular characteristics of afferent discharge regularity or dynamics.

## Anatomy, Synaptic Morphology, and Innervation

Vestibular epithelia of amniotes contain two HC receptor subtypes. These were initially differentiated by HC cytology alone, and were subsequently defined by morphological differences in their associated afferent processes as well (1). Type I HCs are flask-shaped and are innervated by an afferent chalice surrounding the entire basolateral HC surface except the apical neck. The extensiveness and exclusivity of the cleft between the HC and its enveloping calyx, together with the tight junction seal protecting the endolymphatic space, creates a unique, diffusion-limited, femtoliter environment that is markedly different from perilymph. Type II HCs are cylindrical and participate in comparatively small synapses involving bouton-like endings of vestibular afferents that terminate on the receptor somata and the outer calyceal faces (2, 3).

Both vestibular HC subtypes utilize ribbon synapses for quantal transmission. These structures were originally recognized in retinal photoreceptors, where the ribbons were bedecked with synaptic vesicles, a few of which attached to the cell's plasma membrane (4–8). Subsequent studies revealed variability in their overall structure (9, 10), resulting in the adoption of the more general term “synaptic body” to describe synaptic vesicles tethered to a central structure with a minority docked at an active zone.

**Figure 1:**
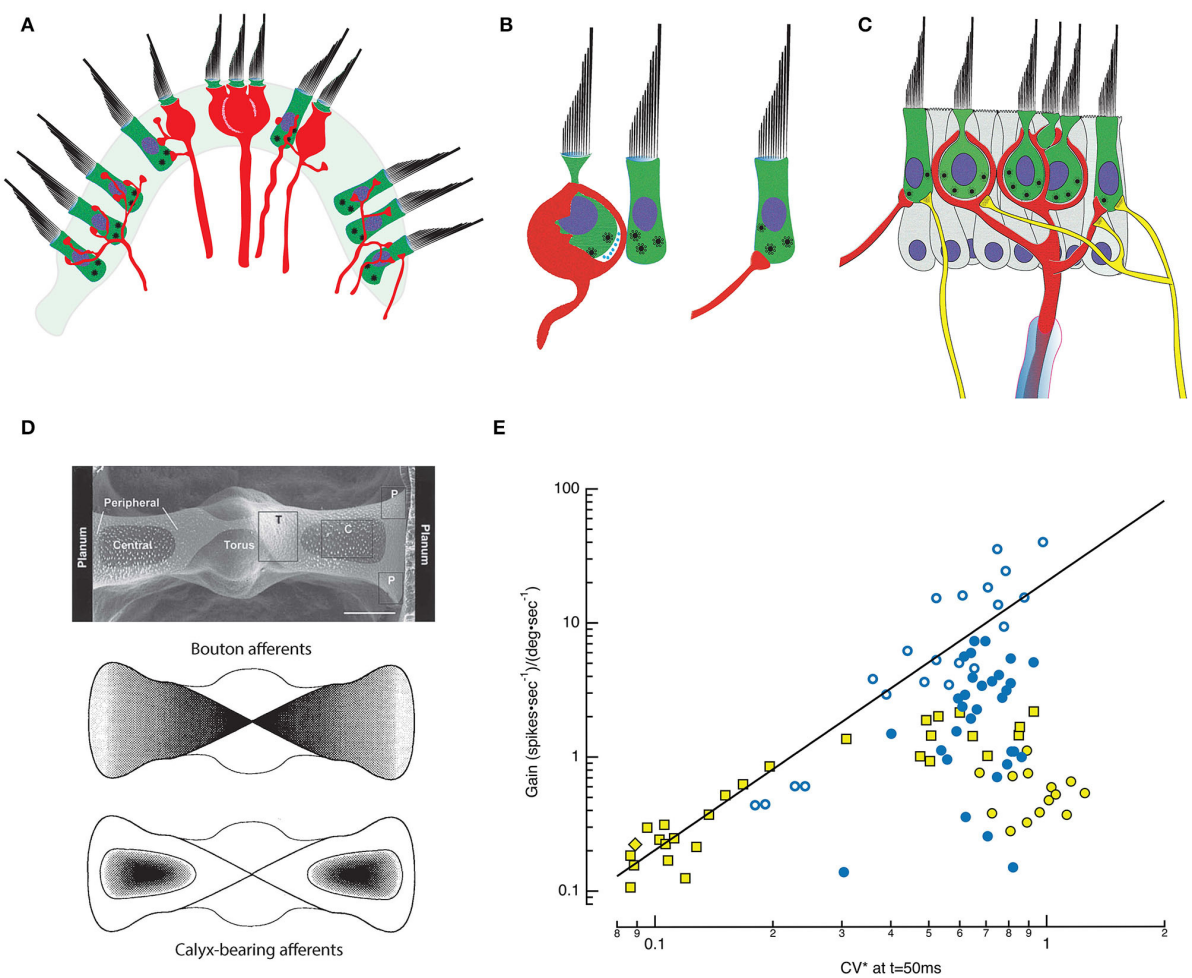
Morphophysiology of the semicircular canal (SCC) epithelium. **(A)** Cross section through the saddle-shaped crista reveals distinct innervation patterns: boutons are found peripherally, with calyx and dimorphic afferents located centrally [adapted from (18)]. **(B)** Type I HCs are internal to an enveloping calyx that creates an extensive synaptic cleft in which ions accumulate. Type I HCs have synaptic bodies associated with quantal transmission onto the inner leaflet of the calyx. Type II HCs also have synaptic bodies indicative of quantal transmission, and synapse onto bouton endings (right) or onto the outer leaflet of an afferent calyx (middle and left). **(C)** A single myelinated afferent (center, red) may branch into several calyces, each containing one or more Type I HCs. Additional input from Type II HCs may occur *via* synapses onto the outer face of the afferent (left), or through bouton endings of fine collateral branches (right). Efferent fibers (yellow) from the brainstem synapse on HCs and on the outer faces of calyces (19). **(D)** Top: SEM plan view of the turtle posterior SCC (20). The epithelium is divided into two hemicristae with central and peripheral regions that extend bilaterally from the torus to the planum on either side. Middle: Map of the density of bouton afferents in each hemicrista. Bottom: Density map of the distribution of calyx-bearing afferents in the central regions of each hemicrista (21). **(E)** Afferent signaling dynamics and discharge statistics in the posterior SCC of chinchilla (yellow symbols) (22) and turtle (blue symbols) (18) are correlated and map systematically across the epithelia. CV*, a measure of the irregularity of the interval between successive APs, is a continuous variable with the most regular fibers on the left and the most irregular on the right. Typically, when either animal was examined with low-frequency, 0.3 or 2 Hz, rotations, low gain afferents sensitive to velocity are more regular, and high gain afferents are sensitive to accelerations. For the chinchilla, the low gain bouton (yellow diamond) and dimorphic (yellow square) afferents demonstrate that regular discharge can be achieved with either bouton or calyx endings. Data seen on the right side, including high gain bouton (turtle: blue, open circle) and dimorphic (turtle: blue, filled circle; chinchilla: yellow square) fibers demonstrate that irregular discharge can be achieved by afferents containing either type of ending. The irregular low gain “calyx only” afferents in chinchilla (yellow filled circle) remain a puzzle but may simply be afferents that respond maximally to stimulations at higher frequencies (23) or with vibration (24). CV* for chinchilla data transformed from 15 ms inter-spike interval (ISI) CV* to that at 50 ms ISI using a third-order polynomial fit to the CV* vs. ISI data at 50 ms in Figure 1 of (22), equivalent to the CV* and ISI used in the turtle data (18).

Synaptic bodies have been reported in retinal photoreceptors and bipolar cells, pinealocytes, electroreceptors, and auditory and vestibular HCs (9). Both the morphology of the synaptic body, and the number, density, tethering, and docking of the associated synaptic vesicles (10, 11) vary. In auditory HCs, this morphological diversity has been correlated with functional differences in peripheral auditory signal processing based on Ca^2+^-dependent exocytosis (12, 13). In both vestibular and auditory HCs, ribbon synapse morphology is thought to underlie the ability of HCs to respond with graded, high rates of sustained quantal transmitter release (14, 15). This dual function is achieved by the orchestrated availability of docked vesicles for immediate exocytosis, and tethered vesicles (the “readily releasable pool”) poised for imminent release (16, 17). As shown in Figure 1, ribbon synapses are present at Type I HC synapses with calyces, and Type II HC synapses with both boutons and calyx outer faces (2, 3, 25, 26). Efferent synapses with afferents and Type II HCs display conventional presynaptic membrane specializations and post-synaptic subsynaptic cisterns in the HCs. Despite this well-defined anatomy, however, the precise role of the calyx in shaping HC-afferent signaling is not known. In that context, it is noteworthy that Wersäll reportedly regretted his sequential naming of the HCs, deeming the Type I HC-calyx architecture more highly evolved (27).

## Morphophysiology

Decades of effort has focused on mapping morphological attributes of vestibular end organs and correlating these maps with dynamics and statistics of afferent firing (28–36); [Review: (37)]. Based on intracellular recordings, dye injections, and light microscopic reconstructions (18, 22), the crista ampullaris in multiple species has been subdivided into concentrically or linearly organized zones that evince differences in HC demographics and afferent innervation patterns (38, 39) (Figure 1D). The posterior canal crista of turtles, for example, is organized into two symmetrical hemicristae separated by a central torus (21, 40). Each hemicrista has a central zone surrounded by a peripheral area that extends toward the torus and planum. All afferents in the central zone have calyces, manifest as either calyx-only fibers (18, 38) or dimorphic (41) fibers having both calyx and bouton endings. Afferents with bouton terminals are present in the peripheral zones throughout the crista, but more densely populate the zone with larger afferent fibers nearest the torus (18). Reflecting this, Type I HCs are more prevalent in the central zone while Type II HCs are present throughout the crista.

This differential localization of HCs and afferent endings across the crista underlies a coherent physiological and functional map of afferent activity (22, 38, 42–46). Specifically, the regularity of afferent fiber discharge, defined by the variability in time between sequential action potentials (APs) is reportedly a continuous variable correlating with the fiber's response to velocity or angular acceleration (47–50). Regular afferents have evenly spaced spontaneous firing rates and respond to low frequency angular motion with tonic discharge. In effect, this mathematically integrates the acceleration stimulus, transmitting signals that are proportional to head velocity. In contrast, the spontaneous firing of irregular afferents shows high variability in the timing between APs. Angular acceleration elicits phasic, dynamic responses from these fibers, which more closely resemble the accelerative stimulus. Moreover, regularly discharging fibers as a group have lower gain (spikes^*^sec^−1^/degrees^*^sec^−1^) than irregular afferents. In turtles, low gain, regularly-firing bouton afferent endings sensitive to velocity are concentrated near the planum, while higher gain bouton afferents sensitive to acceleration terminate closer to the torus (51). In contrast, most calyx-bearing fibers, which end in the central zone, are irregular afferents. The sensitivity of these fibers to low-frequency rotation varies over two orders of magnitude and is not strictly correlated with the degree of irregularity of the spontaneous discharge rate. Nevertheless, an integrated morphophysiological map of the crista emerges in which low gain, regularly discharging, velocity-sensitive bouton afferents terminate nearest the planum, bouton afferents of higher gain and more irregular discharge terminate nearest the torus, and calyx-bearing, irregularly-firing units terminate in the central zone of each hemicrista (Figure 1D). Functionally, the high gain fibers across species are particularly attuned to acceleration (51) or to jerks, the derivative of acceleration (52), and rapidly saturate as the amplitude of the stimulus increases. Medium gain afferents remain active over a wider motion spectrum, while low gain fibers are not prone to saturate, regardless of the magnitude of the head acceleration (Figure 1E). One caveat to this characterization is that the afferent gains were typically assessed at single low frequencies of stimulation (18, 22), although the most irregular fibers are often maximally responsive to higher frequencies and even vibrational or sound stimuli (23, 53, 54).

Based on the initial correlation of afferent terminal distribution with regularity of spontaneous firing and response dynamics, significant effort was directed toward identifying causality. It remained to be determined whether the regularity of a fiber's spontaneous firing derived from the geographical position of its terminals in the crista, or from the biophysical properties of HC inputs to trunk, branch, and twig segments of the parent process. Studies addressing this have been useful in highlighting the overall importance of regional localization in the crista and suggesting the type of HCs, three-dimensional terminal architecture and synapses contributing to individual afferent responses. However, efferent innervation (55–59), GABA-mediated modulation of glutamatergic transmission (60–62), ion accumulation in the synaptic cleft (19, 63–65), and Type II HC input to the external face of the calyx (3, 66) have not been incorporated into these analyses. Since each of these has the potential to markedly alter peripheral vestibular activity, conclusions from these studies are necessarily tentative.

## Solitary Cells

To supplement information gleaned by morphophysiology, currents were analyzed from HCs classified as Type I or II based on their amphoral or cylindrical profile (65, 67–85), from identified regions of the epithelia (20). This approach emulated a strategy used in lower vertebrates to demonstrate that the kinetics of voltage- and ion-sensitive basolateral channels imparted frequency selectivity to solitary auditory HCs (86–92). Typically, solitary vestibular HCs were bathed in artificial perilymph, an ionic environment appropriate for Type II cells, but of unknown propriety for the ionically dynamic milieu of a Type I HC and its calyx. In principle, such a bath would be apposite for isolating conductances based on their voltage ranges of activation, kinetics, ionic permeabilities, and pharmacological profiles. In an artificial perilymph however, analysis of fundamental metrics such as I-V curves would be altered by driving forces reflecting equilibrium potentials for each ion when bathed in high sodium and low potassium. The vestibular potassium channels characterized in this way have been reviewed (93). A startling discovery was that Type I HCs possess a low-voltage outward-rectifying potassium current, IK_(LV)_, that activates at very hyperpolarized potentials. Its full activation near the HC resting potential was considered problematic, since enormous transduction currents would be required to depolarize a cell to potentials necessary for the calcium influx and vesicle fusion associated with quantal transmission. A solution to this paradox was proposed by Chen (94), who suggested that potassium accumulation in the synaptic cleft between the HC and calyx was analogous to the potassium accumulation in tissues surrounding the squid giant axon (95), and that potassium flowing from the HC would increase [K^+^]_cleft_ and depolarize E_K_ for HC and afferent conductances facing the cleft. As a result, the Type I HC would be depolarized, at least to potentials necessary for calcium influx and quantal transmission, but the increased [K^+^]_cleft_ would also depolarize the calyx.

## Theoretical and Single-Electrode Studies

Theoretical and experimental approaches were then used to examine the Chen conjecture. The theoretical analysis (96, 97) relied on equivalent circuits of the Type I HC, cleft, and calyx to analyze the problem primarily as ephaptic transmission (98–100) embellished by potassium accumulation. In general, this analysis concluded that the potential drops associated with current flow along the cleft from HC base to apex created only minor differences in the driving force for channels facing the cleft, and as a result were not likely to be a major source of coupling (97). The possibility that ion accumulation could have the effect suggested by Chen was also examined experimentally (101). Following blockade of quantal transmission, slow potentials that were phase-locked to the stimulus could be detected, a phenomenon consistent with ion accumulation. Two groups examined the potassium accumulation question by perforating the calyx and patch-recording from the enveloped Type I HCs *in situ* (102–104). These experiments demonstrated that depolarization of either the HC or the calyx caused a reversal of the HC potassium tail currents, suggesting an elevation of [K^+^]_cleft_ consistent with Chen's conjecture. Such studies, together with one suggesting a transmitter role for protons (64), yielded results in which the interactions/coupling between cells occurred over tens of milliseconds. However, experiments using mechanical stimulation of the HC bundle demonstrated that when quantal transmission was blocked, the HC-afferent coupling could be extremely rapid, which would facilitate transmission of high frequency information (105, 106). Such contradictory viewpoints evoke the Indian parable of blind men describing an elephant (107), where each detects and interprets a small part of the more complex whole. This led to a quest to discover underlying biophysical mechanisms that could reconcile the differing results.

## Dual-Electrode Studies

The demonstration that [K^+^]_cleft_ can be increased by potassium fluxes from either synaptic partner is a reminder that interpretations of single-electrode voltage-clamp studies of either HC or afferent alone can be problematic. The privileged ionic compartment created by the apposition between a HC and calyx means that any single-electrode study that voltage clamped one cell necessarily allowed the potential of the other cell to vary freely. Moreover, studies that patched onto Type I HCs *in situ* (102–106) did so by perforating the calyx, and there is ample evidence that this approach compromises the cleft environment and results in two types of recordings: those similar to solitary cells bathed in perilymph, and those in which reversal of the potassium tail currents demonstrate an increase in [K^+^]_cleft_ (102, 103).

To address this, we developed an approach to simultaneously patch the HC and calyx without breaching the inner calyceal membrane (19, 63). In essence, this requires patching the HC either on its apex or on the exposed 2–3 μm of its basolateral wall extending apically above the enveloping calyx. With this approach, the potentials of both cells can be interrogated or controlled in a current clamp or voltage clamp, leaving the cleft ionic environment intact.

## Slow Potassium Accumulation Depolarizes Both HC and Afferent to Facilitate Quantal Transmission

**Figure 2:**
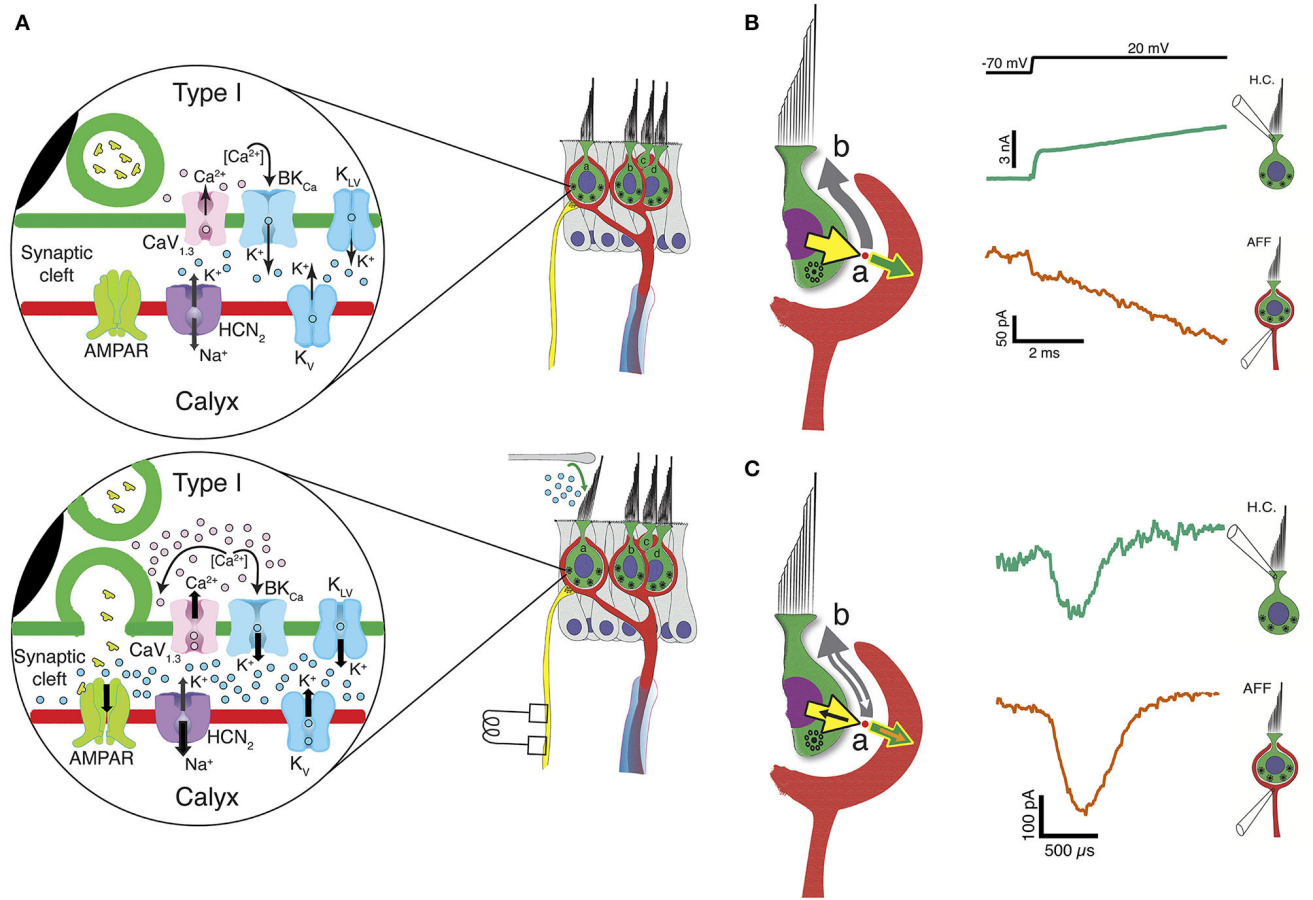
Three forms of synaptic transmission between a Type I HC and calyx afferent. **(A)** In the absence of mechanical stimulation (upper panel), basolateral potassium currents activated at hyperpolarized potentials elevate the [K^+^]_cleft_ to concentrations near 8 mM, twice that found in perilymph. This results in a shift in E_K_ and depolarization of the HC to a resting potential between −60 and −50 mV, where Ca_1, 3_ channels are activated. At rest there is occasional vesicle fusion and the production of a background rate of afferent discharge. Upon mechanical stimulation (lower panel), the inward transduction current flows into the HC and out the basolateral conductances into the cleft as a potassium current. The further increase in [K^+^]_cleft_ is a low-pass integral of the transduction current and further depolarizes E_K_ for conductances in the HC and afferent membranes facing the cleft. The HC depolarization gates Ca^2+^ influx, resulting in fusion of synaptic vesicles and quantal transmission of glutamate to AMPARs on the calyx inner leaflet. The slow accumulation of potassium thereby facilitates quantal transmission near the resting potential, and for maintained depolarizations toward 0 mV, elevates [K^+^]_cleft_ to greater than 28 mM within 25 ms. It is likely that the irregularities of vesicle fusion and large quanta that initiate an AP are driven by neighboring Ca-channel noise (108), and as a consequence the degree of regularity of afferent discharge would be determined by the degree to which the post-synaptic afferent conductances have intrinsic memory and oscillatory behavior similar to those found in HCs of the turtle auditory papilla (90, 109). **(B)** Large conductances in the HC and calyx near the resting potential also create an open resistive pathway between the two that permits a fraction of the transduction current to be communicated across the cleft and into the calyx without synaptic delay. Outward current from the HC (yellow arrow) divides at point (a) into a large component (gray arrow) going along the cleft into the bath at point (b), and a smaller current flowing into the calyx (green arrow). The measured current for the HC (bluish-green current trace) and calyx (vermillion trace) show transmission within 10 μs. **(C)** Resistive coupling can also be demonstrated during large HC depolarizations that depolarize the calyx to potentials sufficient to generate an AP that travels into the calyx. Currents due to the invasion of an AP will sum with the primary currents illustrated in **(B)**. With an AP, an additional inward current (orange arrow) flows into the calyx, with an amplitude equal to the sum of the corresponding currents flowing into the HC (black arrow) and down the cleft (white arrow). The coupling between the calyx retrograde AP current (vermillion) and the induced HC current (bluish-green) is 12 μs [modified from (63)].

Our initial studies (19) focused on ion accumulation and used the reversal potential of the HC potassium tail currents following depolarization to track E_K_ and estimate [K^+^]_cleft_. Blocking HC potassium flux abolished low-pass coupling of the transducer current occurring over tens of milliseconds, but spared quantal transmission. Our subsequent study (63) demonstrated that HC potassium flux depended upon the activation of both voltage-activated and calcium-activated potassium channels, and that the voltage-activated conductance was sensitive to [K^+^]_cleft_ and [Ca^2+^]_cleft_. Clamping [H^+^]_cleft_ resulted in stronger coupling between the two, suggesting that H^+^ was not a primary transmitter acting through acid-sensitive ion channels. Pharmacological experiments using an HCN channel blocker (19) demonstrated that, in addition to synaptic coupling acting through HC and afferent potassium channels, the elevated [K^+^]_cleft_ not only decreased outward K^+^ flux through calyceal voltage-sensitive potassium channels, but also shifted the activation curve of G_HCN_ (84), creating a depolarizing inward flux of Na^+^ and K^+^ through the HCN channels. These potassium-sensitive effects were obtained regardless of whether the increased [K^+^]_cleft_ was due to HC or to afferent fluxes. Inward transduction currents would generate an outward flow of K^+^ through the basolateral HC, elevate [K^+^], and shift E_K_, causing depolarization of both HC and afferent (Figure 2A). Taken together, these results expand Chen's conjecture that potassium accumulation in the cleft facilitates classical quantal transmission between a Type I HC and calyx, and extend it to direct stimulation of the afferent as well. For complex calyces with more than one enveloped HC, the findings indicate that depolarization of one HC can depolarize the afferent, which would then depolarize neighboring receptors enveloped by the shared afferent (Figure 2A). In that light, slow potassium accumulation in the cleft, above that in perilymph, is the ongoing leaky integration of transduction currents or efferent excitation of the calyx, and thus can be interpreted as bidirectional inter-cellular communication that acts over tens of milliseconds.

## HC and Afferent Depolarization Creates Resistive Coupling Through Increased Open-Probability of HC and Afferent Channels Facing the Cleft

One remarkable result of these experiments (63) was a demonstration that there is also a rapid bidirectional coupling between the cells that allows currents to activate within microseconds of depolarization. With depolarization near the HC and calyx resting potentials, there are a significant number of ion channels with high open probabilities. Rapid inward transduction current would immediately flow out as potassium through HC channels, with much of the current flowing into the extensive synaptic cleft and a minority flowing into the calyx (Figure 2B). Such coupling can be analyzed using either Kirchhoff's current or voltage laws, and the conclusion is the same: there would be relatively small voltage gains or drops of a few mV along the cleft depending on the current direction and the precise resistance (97, 110). The impact of this on specific channels facing the cleft is unknown and would greatly depend on their position with respect to the apical gap between HC and calyx. The dual recordings allow us to measure the currents flowing out of one synaptic partner and into the other, or out through the cleft (19, 63) (Figures 2B,C). Such rapid current flow from HC into calyx, Figure 2B, may underlie the rapid transmission of high frequency mechanical stimuli (105, 106, 111), and may be the substrate for detection of sound and vibration (24, 33, 54, 112–117). As with slow potassium accumulation, this rapid ion flow through open channels is bidirectional, as evidenced by the retrograde transmission of calyx afferent APs into the HC (Figure 2C) (19). Thus, as depolarization persists, [K^+^]_cleft_ increases, and both the number of open channels and the amount of HC-afferent electrical coupling increase concomitantly.

Dual recordings also provided the incidental observation that in the central region, the EPSCs from Type I HCs onto the calyx inner leaflet were small (19, 118, 119), whereas those from Type II HCs onto the outer leaflet (19) were as large as those reported in the cochlea (14), and would be sufficient to trigger APs in afferents near the spike threshold. This highlights the risk of morphophysiological studies defining calyx-only afferents based on light microscopy alone.

## Conclusions

During vertebrate evolution, selective pressures favored increased sensitivity and flawless encoding of three-dimensional vestibular information. This correlated with a higher prevalence of Type I HCs and calyx afferents (120–123), which exhibit unidirectional quantal and two forms of bidirectional communication. Potassium accumulation in the synaptic cleft is a low-pass filter of HC transduction currents and afferent depolarizations by efferent fibers, and thus a leaky integrator of activity. At rest, [K^+^]_cleft_ is roughly 8 mM, or twice that found in perilymph, and is sufficient to depolarize E_K_ and facilitate conventional quantal transmission. Maintained low-frequency excitation elevates [K^+^]_cleft_ further to >28 mM, a value sufficient to trigger afferent APs even in the absence of quantal transmission. From an evolutionary perspective, the pressure to extend transmission to higher frequencies was best served by direct resistive coupling created by large HC and afferent cleft-facing conductances present at and depolarized from rest. In evolving from bouton, through protocalyx to calyx, these conductances elevate [K^+^]_cleft_, depolarize E_K_ to facilitate quantal transmission, and provide a resistive path between synaptic partners that has minimal delay, is dynamic, and would increase as mechanical inputs become larger.

## Author Contributions

All authors listed have made a substantial, direct, and intellectual contribution to the work and approved it for publication.

## Funding

This work was supported by NIH National Institute on Deafness and Other Communication Disorders grants R21 DC017292; R21 DC017577, R01 DC019953, and R01 DC008846.

## Conflict of Interest

The authors declare that the research was conducted in the absence of any commercial or financial relationships that could be construed as a potential conflict of interest.

## Publisher's Note

All claims expressed in this article are solely those of the authors and do not necessarily represent those of their affiliated organizations, or those of the publisher, the editors and the reviewers. Any product that may be evaluated in this article, or claim that may be made by its manufacturer, is not guaranteed or endorsed by the publisher.

## References

[1] Wersäll J. Studies on the structure and innervation of the sensory epithelium of the cristae ampullares in the guinea pig. Acta Otolaryngol Suppl. (1956) 126:7–85. 10.3109/00016485609120818 13326368

[2] Goldberg JM Lysakowski A Fernández C. Morphophysiological and ultrastructural studies in the mammalian cristae ampullares. Hear Res. (1990) 49:89–102. 10.1016/0378-5955(90)90097-9 2292511

[3] Lysakowski A Goldberg JM. Ultrastructural analysis of the cristae ampullares in the squirrel monkey (*Saimiri sciureus*). J Comp Neurol. (2008) 511:47–64. 10.1002/cne.21827 18729176 PMC2828494

[4] Sjöstrand FS editor. The ultrastructure of the innersegments of the retinal rods of the guinea pig eye as revealed by electron microscopy. J Appl Phys. (1953) 42:45–70. 10.1002/jcp.1030420104 13084706

[5] De Robertis E Franchi CM. Electron microscope observations on synaptic vesicles in synapses of the retinal rods and cones. J Biophys Biochem Cytol. (1956) 2:307–18. 10.1083/jcb.2.3.307 13331963 PMC2223974

[6] Carasso N. Etude au microscope électronique des synapses des cellules visuelles chez le têtard d'Alytes obstetricans. [Electron microscopy of the synapses of the visual cells in the tadpole of Alytes obstetricans]. Comptes Rendus Hebdomadaires Séances Acad Sci D Sci Nat. (1957) 245:216–9. 13461383

[7] Polyak SL. The Retina. Chicago, IL: The University of Chicago Press (1941).

[8] Sjostrand FS. Ultrastructure of retinal rod synapses of the guinea pig eye as revealed by three-dimensional reconstructions from serial sections. J Ultrastruct Res. (1958) 2:122–70. 10.1016/S0022-5320(58)90050-9 13631744

[9] Sterling P Matthews G. Structure and function of ribbon synapses. Trends Neurosci. (2005) 28:20–9. 10.1016/j.tins.2004.11.009 15626493

[10] Moser T Brandt A Lysakowski A. Hair cell ribbon synapses. Cell Tissue Res. (2006) 326:347–59. 10.1007/s00441-006-0276-3 16944206 PMC4142044

[11] Schmitz F. The making of synaptic ribbons: how they are built and what they do. Neuroscientist. (2009) 15:611–24. 10.1177/1073858409340253 19700740

[12] Johnson SL Forge A Knipper M Munkner S Marcotti W. Tonotopic variation in the calcium dependence of neurotransmitter release and vesicle pool replenishment at mammalian auditory ribbon synapses. J Neurosci. (2008) 28:7670–8. 10.1523/JNEUROSCI.0785-08.2008 18650343 PMC2516938

[13] Meyer AC Frank T Khimich D Hoch G Riedel D Chapochnikov NM. Tuning of synapse number, structure and function in the cochlea. Nature Neurosci. (2009) 12:444–53. 10.1038/nn.2293 19270686

[14] Glowatzki E Fuchs PA. Transmitter release at the hair cell ribbon synapse. Nat Neurosci. (2002) 5:147–54. 10.1038/nn796 11802170

[15] Fuchs PA Glowatzki E Moser T. The afferent synapse of cochlear hair cells. Curr Opin Neurobiol. (2003) 13:452–8. 10.1016/S0959-4388(03)00098-9 12965293

[16] von Gersdorff H Matthews G. Electrophysiology of synaptic vesicle cycling. Annu Rev Physiol. (1999) 61:725–52. 10.1146/annurev.physiol.61.1.725 10099708

[17] Holt M Cooke A Neef A Lagnado L. High mobility of vesicles supports continuous exocytosis at a ribbon synapse. Curr Biol. (2004) 14:173–83. 10.1016/j.cub.2003.12.053 14761649

[18] Brichta AM Goldberg JM. Morphological identification of physiologically characterized afferents innervating the turtle posterior crista. J Neurophysiol. (2000) 83:1202–23. 10.1152/jn.2000.83.3.1202 10712450

[19] Contini D Price SD Art JJ. Accumulation of K+ in the synaptic cleft modulates activity by influencing both vestibular hair cell and calyx afferent in the turtle. J Physiol. (2017) 595.3:777–803. 10.1113/JP273060 27633787 PMC5285615

[20] Brichta AM Aubert A Eatock RA Goldberg JM. Regional analysis of whole cell currents from hair cells of the turtle posterior crista. J Neurophysiol. (2002) 88:3259–78. 10.1152/jn.00770.2001 12466445

[21] Brichta AM Peterson EH. Functional architecture of vestibular primary afferents from the posterior semicircular canal of a turtle, *Pseudemys (Trachemys) scripta* elegans. J Comp Neurol. (1994) 344:481–507. 10.1002/cne.903440402 7929889

[22] Baird RA Desmadryl G Fernández C Goldberg JM. The vestibular nerve of the chinchilla. II. Relation between afferent response properties and peripheral innervation patterns in the semicircular canals. J Neurophysiol. (1988) 60:182–203. 10.1152/jn.1988.60.1.182 3404216

[23] Dickman JD Correia MJ. Responses of pigeon horizontal semicircular canal afferent fibers II. High-frequency mechanical stimulation. J Neurophysiol. (1989) 62:1102–12. 10.1152/jn.1989.62.5.1102 2585042

[24] Curthoys IS Grant JW. How does high-frequency sound or vibration activate vestibular receptors? Exp Brain Res. (2015) 233:691–9. 10.1007/s00221-014-4192-6 25567092

[25] Lysakowski A Goldberg JM. A regional ultrastructural analysis of the cellular and synaptic architecture in the chinchilla cristae ampullares. J Comp Neurol. (1997) 389:419–43. 9414004 10.1002/(sici)1096-9861(19971222)389:3<419::aid-cne5>3.0.co;2-3 PMC11468975

[26] Lysakowski A. Synaptic organization of the crista ampullaris in vertebrates. Ann N Y Acad Sci. (1996) 781:164–82. 10.1111/j.1749-6632.1996.tb15700.x 8694413

[27] Engström H. The first-order vestibular neuron. In: Graybiel A, editor. Symposium on the Role of the Vestibular Organs in Space Exploration NASA SP-187. Washington, DC: National Academies (1970). p. 123–35.

[28] Goldberg JM Fernández C. Vestibular mechanisms. Annu Rev Physiol. (1975) 37:129–62. 10.1146/annurev.ph.37.030175.001021 164818

[29] Suzuki M Harada Y. An experimental study on the frog semicircular canal–functions of cupula and vestibular ganglion. J Otolaryngol. (1985) 14:36–40. 3877815

[30] Suzuki M Harada Y. An experimental study on the physiological significance of the mode of cupular movement. Arch Otorhinolaryngol. (1985) 242:57–62. 10.1007/BF00464407 3876091

[31] Rabbitt RD Boyle R Highstein SM. Sensory transduction of head velocity and acceleration in the toadfish horizontal semicircular canal. J Neurophysiol. (1994) 72:1041–8. 10.1152/jn.1994.72.2.1041 7983512

[32] Hullar TE Della Santina CC Hirvonen T Lasker DM Carey JP Minor LB. Responses of irregularly discharging chinchilla semicircular canal vestibular-nerve afferents during high-frequency head rotations. J Neurophysiol. (2005) 93:2777–86. 10.1152/jn.01002.2004 15601735

[33] Curthoys IS Kim J McPhedran SK Camp AJ. Bone conducted vibration selectively activates irregular primary otolithic vestibular neurons in the guinea pig. Exp Brain Res. (2006) 175:256–67. 10.1007/s00221-006-0544-1 16761136

[34] Lasker DM Han GC Park HJ Minor LB. Rotational responses of vestibular-nerve afferents innervating the semicircular canals in the C57BL/6 mouse. J Assoc Res Otolaryngol. (2008) 9:334–48. 10.1007/s10162-008-0120-4 18473139 PMC2538153

[35] Jamali M Sadeghi SG Cullen KE. Response of vestibular nerve afferents innervating utricle and saccule during passive and active translations. J Neurophysiol. (2009) 101:141–9. 10.1152/jn.91066.2008 18971293 PMC3815216

[36] Yu XJ Dickman JD Angelaki DE. Detection thresholds of macaque otolith afferents. J Neurosci. (2012) 32:8306–16. 10.1523/JNEUROSCI.1067-12.2012 22699911 PMC3403680

[37] Paulin MG Hoffman LF. Models of vestibular semicircular canal afferent neuron firing activity. J Neurophysiol. (2019) 122:2548–67. 10.1152/jn.00087.2019 31693427 PMC6966309

[38] Fernández C Baird RA Goldberg JM. The vestibular nerve of the chinchilla. I. Peripheral innervation patterns in the horizontal and superior semicircular canals. J Neurophysiol. (1988) 60:167–81. 10.1152/jn.1988.60.1.167 3404215

[39] Fernandez C Lysakowski A Goldberg JM. Hair-cell counts and afferent innervation patterns in the cristae ampullares of the squirrel monkey with a comparison to the chinchilla. J Neurophysiol. (1995) 73:1253–69. 10.1152/jn.1995.73.3.1253 7608769

[40] Brichta AM Peterson EH. Vestibular afferents innervating the posterior ampullae in a turtle, *Pseudemys scripta*. Ann N Y Acad Sci. (1992) 656:914–6. 10.1111/j.1749-6632.1992.tb25293.x 1599219

[41] Schessel DA Highstein SM. Is transmission between the vestibular type I hair cell and its primary afferent chemical? Ann N Y Acad Sci. (1981) 374:210–4. 10.1111/j.1749-6632.1981.tb30871.x 6122418

[42] Schessel DA Ginzberg R Highstein SM. Morphophysiology of synaptic transmission between type I hair cells and vestibular primary afferents. An intracellular study employing horseradish peroxidase in the lizard, Calotes versicolor. Brain Res. (1991) 544:1–16. 10.1016/0006-8993(91)90879-Z 1713111

[43] Goldberg JM Baird RA Fernández C. Morphophysiological studies of the mammalian vestibular labyrinth. Prog Clin Biol Res. (1985) 176:231–45. 2987974

[44] Fernández C Goldberg JM Baird RA. The vestibular nerve of the chinchilla. III. Peripheral innervation patterns in the utricular macula. J Neurophysiol. (1990) 63:767–80. 10.1152/jn.1990.63.4.767 2341875

[45] Goldberg JM Desmadryl G Baird RA Fernández C. The vestibular nerve of the chinchilla. V. Relation between afferent discharge properties and peripheral innervation patterns in the utricular macula. J Neurophysiol. (1990) 63:791–804. 10.1152/jn.1990.63.4.791 2341877

[46] Goldberg JM Desmadryl G Baird RA Fernández C. The vestibular nerve of the chinchilla. IV. Discharge properties of utricular afferents. J Neurophysiol. (1990) 63:781–90. 10.1152/jn.1990.63.4.781 2341876

[47] Fernández C Goldberg JM. Physiology of peripheral neurons innervating semicircular canals of the squirrel monkey. II. Response to sinusoidal stimulation and dynamics of peripheral vestibular system. J Neurophysiol. (1971) 34:661–75. 10.1152/jn.1971.34.4.661 5000363

[48] Goldberg JM Fernández C. Physiology of peripheral neurons innervating semicircular canals of the squirrel monkey. III. Variations among units in their discharge properties. J Neurophysiol. (1971) 34:676–84. 10.1152/jn.1971.34.4.676 5000364

[49] Goldberg JM Fernández C. Physiology of peripheral neurons innervating semicircular canals of the squirrel monkey. I. Resting discharge and response to constant angular accelerations. J Neurophysiol. (1971) 34:635–60. 5000362 10.1152/jn.1971.34.4.635

[50] Curthoys IS. The response of primary horizontal semicircular canal neurons in the rat and guinea pig to angular acceleration. Exp Brain Res. (1982) 47:286–94. 10.1007/BF00239388 7117453

[51] Brichta AM Goldberg JM. Responses to efferent activation and excitatory response-intensity relations of turtle posterior-crista afferents. J Neurophysiol. (2000) 83:1224–42. 10.1152/jn.2000.83.3.1224 10712451

[52] Art JJ. Comparative physiology of primary afferents innervating semicircular canals in two species of plethodontid salamanders (Dissertation). Chicago, IL: University of Chicago (1979).

[53] McCue MP Guinan JJ Jr. Acoustically responsive fibers in the vestibular nerve of the cat. J Neurosci. (1994) 14:6058–70. 10.1523/JNEUROSCI.14-10-06058.1994 7931562 PMC6576982

[54] Curthoys IS Burgess AM Goonetilleke SC. Phase-locking of irregular guinea pig primary vestibular afferents to high frequency (>250Hz) sound and vibration. Hear Res. (2019) 373:59–70. 10.1016/j.heares.2018.12.009 30599427

[55] Poppi LA Holt JC Lim R Brichta AM. A review of efferent cholinergic synaptic transmission in the vestibular periphery and its functional implications. J Neurophysiol. (2019). 10.1152/jn.00053.2019 31800345 PMC7132328

[56] Holt JC Jordan PM Lysakowski A Shah A Barsz K Contini D. Muscarinic acetylcholine receptors and m-currents underlie efferent-mediated slow excitation in calyx-bearing vestibular afferents. J Neurosci. (2017) 37:1873–87. 10.1523/JNEUROSCI.2322-16.2017 28093476 PMC5320615

[57] Holt JC Kewin K Jordan PM Cameron P Klapczynski M McIntosh JM. Pharmacologically distinct nicotinic acetylcholine receptors drive efferent-mediated excitation in calyx-bearing vestibular afferents. J Neurosci. (2015) 35:3625–43. 10.1523/JNEUROSCI.3388-14.2015 25716861 PMC4339364

[58] Jordan PM Parks XX Contini D Holt JC. A review of synaptic mechanisms of vestibular efferent signaling in turtles: extrapolation to efferent actions in mammals. J Vestib Res. (2013) 23:161–75. 10.3233/VES-130492 24177348

[59] Raghu V Salvi R Sadeghi SG. Efferent inputs are required for normal function of vestibular nerve afferents. J Neurosci. (2019) 39:6922–35. 10.1523/JNEUROSCI.0237-19.2019 31285300 PMC6733555

[60] Holstein GR Rabbitt RD Martinelli GP Friedrich VL Jr Boyle RD Highstein SM Convergence of excitatory and inhibitory hair cell transmitters shapes vestibular afferent responses. Proc Natl Acad Sci USA. (2004) 101:15766–71. 10.1073/pnas.0402824101 15505229 PMC524830

[61] Holstein GR Martinelli GP Henderson SC Friedrich VL Jr Rabbitt RD Highstein SM. Gamma-aminobutyric acid is present in a spatially discrete subpopulation of hair cells in the crista ampullaris of the toadfish *Opsanus tau*. J Comp Neurol. (2004) 471:1–10. 10.1002/cne.11025 14983471

[62] Highstein SM Rabbitt RD Holstein GR Boyle RD. Determinants of spatial and temporal coding by semicircular canal afferents. J Neurophysiol. (2005) 93:2359–70. 10.1152/jn.00533.2004 15845995 PMC3000935

[63] Contini D Holstein GR Art JJ. Synaptic cleft microenvironment influences potassium permeation and synaptic transmission in hair cells surrounded by calyx afferents in the turtle. J Physiology. (2020) 598.4:853–89. 10.1113/JP278680 31623011 PMC7024053

[64] Highstein SM Holstein GR Mann MA Rabbitt RD. Evidence that protons act as neurotransmitters at vestibular hair cell-calyx afferent synapses. Proc Natl Acad Sci USA. (2014) 111:5421–6. 10.1073/pnas.1319561111 24706862 PMC3986198

[65] Chen JW Eatock RA. Major potassium conductance in type I hair cells from rat semicircular canals: characterization and modulation by nitric oxide. J Neurophysiol. (2000) 84:139–51. 10.1152/jn.2000.84.1.139 10899192

[66] Ross MD Rogers CM Donovan KM. Innervation patterns in rat saccular macula. A structural basis for complex sensory processing. Acta Otolaryngol. (1986) 102:75–86. 10.3109/00016488609108649 2943113

[67] Valat J Devau G Dulon D Sans A. Vestibular hair cells isolated from guinea pig labyrinth. Hear Res. (1989) 40:255–60. 10.1016/0378-5955(89)90166-4 2793607

[68] Masetto S Russo G Taglietti V Prigioni I. [K+ and Ca++ currents in hair cells isolated from the semicircular canals of the frog]. Boll Soc Ital Biol Sper. (1991) 67:493–500. 1666831

[69] Rennie KJ Ashmore JF. Ionic currents in isolated vestibular hair cells from the guinea-pig crista ampullaris. Hear Res. (1991) 51:279–92. 10.1016/0378-5955(91)90044-A 2032962

[70] Prigioni I Masetto S Russo G Taglietti V. Calcium currents in solitary hair cells isolated from frog crista ampullaris. J Vestib Res. (1992) 2:31–9. 10.3233/VES-1992-2103 1285264

[71] Griguer C Kros CJ Sans A Lehouelleur J. Potassium currents in type II vestibular hair cells isolated from the guinea-pig's crista ampullaris [published erratum appears in Pflugers Arch 1994 May;427:193]. Pflugers Archiv Eur J Physiol. (1993) 425:344–52. 10.1007/BF00374185 8060388

[72] Griguer C Sans A Lehouelleur J. Non-typical K(+)-current in cesium-loaded guinea pig type I vestibular hair cell. Pflugers Archiv Eur J Physiol. (1993) 422:407–9. 10.1007/BF00374300 8437892

[73] Griguer C Sans A Valmier J Lehouelleur J. Inward potassium rectifier current in type I vestibular hair cells isolated from guinea pig. Neurosci Lett. (1993) 149:51–5. 10.1016/0304-3940(93)90345-L 8469379

[74] Lapeyre PN Kolston PJ Ashmore JF. GABAB-mediated modulation of ionic conductances in type I hair cells isolated from guinea-pig semicircular canals. Brain Res. (1993) 609:269–76. 10.1016/0006-8993(93)90882-N 7685230

[75] Rennie KJ Ashmore JF. Effects of extracellular ATP on hair cells isolated from the guinea-pig semicircular canals. Neurosci Lett. (1993) 160:185–9. 10.1016/0304-3940(93)90409-E 8247351

[76] Rennie KJ Correia MJ. Potassium currents in mammalian and avian isolated type I semicircular canal hair cells. J Neurophysiol. (1994) 71:317–29. 10.1152/jn.1994.71.1.317 8158233

[77] Ricci AJ Cochran SL Rennie KJ Correia MJ. Vestibular type I and type II hair cells. 2: morphometric comparisons of dissociated pigeon hair cells. J Vestib Res. (1997) 7:407–20. 10.3233/VES-1997-7504 9376914

[78] Ricci AJ Rennie KJ Cochran SL Kevetter GA Correia MJ. Vestibular type I and type II hair cells. 1: morphometric identification in the pigeon and gerbil. J Vestib Res. (1997) 7:393–406. 10.3233/VES-1997-7503 9376913

[79] Eatock RA Rusch A Lysakowski A Saeki M. Hair cells in mammalian utricles. Otolaryngol Head Neck Surg. (1998) 119:172–81. 10.1016/S0194-5998(98)70052-X 9743073

[80] Marcotti W Russo G Prigioni I. Inactivating and non-activating delayed rectifier K+ currents in hair cells of frog crista ampullaris. Hear Res. (1999) 135:113–23. 10.1016/S0378-5955(99)00097-0 10491960

[81] Martini M Rossi ML Rubbini G Rispoli G. Calcium currents in hair cells isolated from semicircular canals of the frog. Biophys J. (2000) 78:1240–54. 10.1016/S0006-3495(00)76681-1 10692313 PMC1300726

[82] Rennie KJ Weng T Correia MJ. Effects of KCNQ channel blockers on K(+) currents in vestibular hair cells. Am J Physiol Cell Physiol. (2001) 280:C473–80. 10.1152/ajpcell.2001.280.3.C473 11171566

[83] Li GQ Meredith FL Rennie KJ. Development of K(+) and Na(+) conductances in rodent postnatal semicircular canal type I hair cells. Am J Physiol Regul Integr Comp Physiol. (2010) 298:R351–8. 10.1152/ajpregu.00460.2009 19939976 PMC2828173

[84] Meredith FL Benke TA Rennie KJ. Hyperpolarization-activated current. (I(h)) in vestibular calyx terminals: characterization and role in shaping postsynaptic events. J Assoc Res Otolaryngol. (2012) 13:745–58. 10.1007/s10162-012-0342-3 22825486 PMC3505587

[85] Mann SE Johnson M Meredith FL Rennie KJ. Inhibition of K+ currents in type I vestibular hair cells by gentamicin and neomycin. Audiol Neurootol. (2013) 18:317–26. 10.1159/000354056 24051519

[86] Lewis RS Hudspeth AJ. Voltage- and ion-dependent conductances in solitary vertebrate hair cells. Nature. (1983) 304:538–41. 10.1038/304538a0 6603579

[87] Lewis RS Hudspeth AJ. Frequency tuning and ionic conductances in hair cells of the bullfrog's sacculus. In: Klinke R, Hartmann R, editors. Hearing-Physiological Bases and Psychophysics. Berlin: Springer-Verlag (1983). p. 17–24. 10.1007/978-3-642-69257-4_3

[88] Hudspeth AJ Lewis RS. A model for electrical resonance and frequency tuning in saccular hair cells of the bull-frog, *Rana catesbeiana*. J Physiol. (1988) 400:275–97. 10.1113/jphysiol.1988.sp017120 2458455 PMC1191807

[89] Hudspeth AJ Lewis RS. Kinetic analysis of voltage- and ion-dependent conductances in saccular hair cells of the bull-frog, Rana catesbeiana. J Physiol. (1988) 400:237–74. 10.1113/jphysiol.1988.sp017119 2458454 PMC1191806

[90] Art JJ Fettiplace R. Variation of membrane properties in hair cells isolated from the turtle cochlea. J Physiol. (1987) 385:207–42. 10.1113/jphysiol.1987.sp016492 2443666 PMC1192345

[91] Art JJ Crawford AC Fettiplace R. Electrical resonance and membrane currents in turtle cochlear hair cells. Hear Res. (1986) 22:31–6. 10.1016/0378-5955(86)90073-0 2426237

[92] Smotherman MS Narins PM. The electrical properties of auditory hair cells in the frog amphibian papilla. J Neurosci. (1999) 19:5275–92. 10.1523/JNEUROSCI.19-13-05275.1999 10377339 PMC6782313

[93] Meredith FL Rennie KJ. Channeling your inner ear potassium: K(+) channels in vestibular hair cells. Hear Res. (2016). 10.1016/j.heares.2016.01.015 26836968

[94] Chen W-Y. The properties and functions of a low-voltage activated K(+) current in type I hair cells of rat semicircular canal organs (Dissertation). Rochester, NY: University of Rochester (1995).

[95] Frankenhaeuser B Hodgkin AL. The after-effects of impulses in the giant nerve fibres of Loligo. J Physiol. (1956) 131:341–76. 10.1113/jphysiol.1956.sp005467 13320339 PMC1363434

[96] Goldberg JM. Transmission between the type I hair cell and its calyx ending. Ann N Y Acad Sci N Direct Vestib Res. (1996) 781:474–88. 10.1111/j.1749-6632.1996.tb15721.x 8694437

[97] Goldberg JM. Theoretical analysis of intercellular communication between the vestibular type I hair cell and its calyx ending. J Neurophysiol. (1996) 76:1942–57. 10.1152/jn.1996.76.3.1942 8890305

[98] Arvanitaki A. Effects evoked in an axon by the activity of a contiguous one. J Neurophysiol. (1942) 5:89–108. 10.1152/jn.1942.5.2.89

[99] Katz B Schmitt OH. Electric interaction between two adjacent nerve fibres. J Physiol. (1940) 97:471–88. 10.1113/jphysiol.1940.sp003823 16995178 PMC1393925

[100] Kramer RH Davenport CM. Lateral inhibition in the vertebrate retina: the case of the missing neurotransmitter. PLoS Biol. (2015) 13:e1002322. 10.1371/journal.pbio.1002322 26656622 PMC4675548

[101] Holt JC Chatlani S Lysakowski A Goldberg JM. Quantal and nonquantal transmission in calyx-bearing fibers of the turtle posterior crista. J Neurophysiol. (2007) 98:1083–101. 10.1152/jn.00332.2007 17596419 PMC3397384

[102] Contini D. Intercellular K+ accumulation drives Type I hair cells into the activation range of voltage-gated Ca channels (Dissertation). Pavia, Italy: University of Pavia (2011).

[103] Contini D Zampini V Tavazzani E Magistretti J Russo G Prigioni I. Intercellular K(+) accumulation depolarizes Type I vestibular hair cells and their associated afferent nerve calyx. Neuroscience. (2012) 227:232–46. 10.1016/j.neuroscience.2012.09.051 23032932

[104] Lim R Kindig AE Donne SW Callister RJ Brichta AM. Potassium accumulation between type I hair cells and calyx terminals in mouse crista. Exp Brain Res. (2011) 210:607–21. 10.1007/s00221-011-2592-4 21350807

[105] Eatock RA Songer JE. Vestibular hair cells and afferents: two channels for head motion signals. Annu Rev Neurosci. (2011) 34:501–34. 10.1146/annurev-neuro-061010-113710 21469959

[106] Songer JE Eatock RA. Tuning and timing in mammalian type I hair cells and calyceal synapses. J Neurosci. (2013) 33:3706–24. 10.1523/JNEUROSCI.4067-12.2013 23426697 PMC3857958

[107] Ireland J. The Udana & The Itivuttaka: Inspired Utterances of the Buddha & The Buddha's Sayings. Kandy: BPS Pariyatti Editions (2020).

[108] Ramachandran S Rodgriguez S Potcoava M Alford S. Single calcium channel nanodomains drive presynaptic calcium entry at lamprey reticulospinal presynaptic terminals. J Neurosci. (2022) 42:2385–403. 10.1523/JNEUROSCI.2207-21.2022 35063999 PMC8944239

[109] Art JJ Wu YC Fettiplace R. The calcium-activated potassium channels of turtle hair cells. J Gen Physiol. (1995) 105:49–72. 10.1085/jgp.105.1.49 7730789 PMC2216930

[110] Govindaraju AC Quraishi IH Lysakowski A Eatock RA Raphael RM. A Biophysical model of nonquantal transmission at the vestibular hair cell-calyx synapse: K(LV) currents modulate fast electrical and slow K(+) potentials in the synaptic cleft. bioRxiv. (2021):2021.11.18.469197. 10.1101/2021.11.18.469197

[111] Eatock RA. Specializations for fast signaling in the amniote vestibular inner ear. Integr Comp Biol. (2018) 58:341–50. 10.1093/icb/icy069 29920589 PMC6104706

[112] McCue MP Guinan JJ Jr. Sound-evoked activity in primary afferent neurons of a mammalian vestibular system. Am J Otol. (1997) 18:355–60. 9149831

[113] McCue MP Guinan JJ Jr. Spontaneous activity and frequency selectivity of acoustically responsive vestibular afferents in the cat. J Neurophysiol. (1995) 74:1563–72. 10.1152/jn.1995.74.4.1563 8989393

[114] McCue MP Guinan JJ Jr. Influence of efferent stimulation on acoustically responsive vestibular afferents in the cat. J Neurosci. (1994) 14:6071–83. 10.1523/JNEUROSCI.14-10-06071.1994 7931563 PMC6577006

[115] Curthoys IS Vulovic V. Vestibular primary afferent responses to sound and vibration in the guinea pig. Exp Brain Res. (2011) 210:347–52. 10.1007/s00221-010-2499-5 21113779

[116] Curthoys IS Vulovic V Sokolic L Pogson J Burgess AM. Irregular primary otolith afferents from the guinea pig utricular and saccular maculae respond to both bone conducted vibration and to air conducted sound. Brain Res Bull. (2012) 89:16–21. 10.1016/j.brainresbull.2012.07.007 22814095

[117] Curthoys IS Vulovic V Burgess AM Sokolic L Goonetilleke SC. The response of guinea pig primary utricular and saccular irregular neurons to bone-conducted vibration (BCV) and air-conducted sound (ACS). Hear Res. (2016) 331:131–43. 10.1016/j.heares.2015.10.019 26626360

[118] Highstein SM Mann MA Holstein GR Rabbitt RD. The quantal component of synaptic transmission from sensory hair cells to the vestibular calyx. J Neurophysiol. (2015) 113:3827–35. 10.1152/jn.00055.2015 25878150 PMC4473518

[119] Sadeghi SG Pyott SJ Yu Z Glowatzki E. Glutamatergic signaling at the vestibular hair cell calyx synapse. J Neurosci. (2014) 34:14536–50. 10.1523/JNEUROSCI.0369-13.2014 25355208 PMC4212060

[120] Lysakowski A Minor LB Fernandez C Goldberg JM. Physiological identification of morphologically distinct afferent classes innervating the cristae ampullares of the squirrel monkey. J Neurophysiol. (1995) 73:1270–81. 7608770 10.1152/jn.1995.73.3.1270

[121] Kirkegaard M Jorgensen JM. The inner ear macular sensory epithelia of the Daubenton's bat. J Comp Neurol. (2001) 438:433–44. 11559899 10.1002/cne.1326

[122] Jorgensen JM. Number and distribution of hair cells in the utricular macula of some avian species. J Morphol. (1989) 201:187–204. 10.1002/jmor.1052010208 29865667

[123] Jorgensen JM Christensen JT. The inner ear of the common rhea (Rhea americana L). Brain Behav Evol. (1989) 34:273–80. 10.1159/000116512 2575429

